# Long-Term Outcomes of Hepatic Resection versus Living Donor Liver Transplantation for Hepatocellular Carcinoma: A Propensity Score-Matching Study

**DOI:** 10.1155/2015/425926

**Published:** 2015-04-01

**Authors:** Toshimi Kaido, Satoshi Morita, Sachiko Tanaka, Kohei Ogawa, Akira Mori, Etsuro Hatano, Shinji Uemoto

**Affiliations:** ^1^Division of Hepato-Biliary-Pancreatic and Transplant Surgery, Department of Surgery, Graduate School of Medicine, Kyoto University, Kyoto 606-8507, Japan; ^2^Clinical Research Center for Medical Equipment Development, Kyoto University Hospital, Kyoto 606-8507, Japan; ^3^Department of Pharmacoepidemiology, Graduate School of Medicine and Public Health, Kyoto University, Kyoto 606-8507, Japan

## Abstract

Hepatic resection (HR) and liver transplantation (LT) are surgical treatment options for hepatocellular carcinoma (HCC). However, it is clinically impossible to perform a randomized, controlled study to determine the usefulness of these treatments. The present study compared survival rates and recurrence rates of HR versus living donor LT (LDLT) for HCC by using the propensity score method. Between January 1999 and August 2012, 936 patients (732 HR, 204 LDLT) underwent surgical therapy for HCC in our center. Using the propensity score matching, 80 well-balanced patients were defined. The 1- and 5-year overall survival rates were 90% and 53% in the HR group and 82% and 63% in the LT group, respectively. They were not significantly different between the two groups. The odds ratio estimated using the propensity score matching analysis was 0.842 (*P* = 0.613). The 1- and 5-year recurrence rates were significantly lower in the LT group (9% and 21%) than in the HR group (43% and 74%) (*P* < 0.001), and the odds ratio was 0.214 (*P* = 0.001). In conclusion, HR should be considered a valid alternative to LDLT taking into consideration the risk for the living donor based on the results of this propensity score-matching study.

## 1. Introduction

Hepatocellular carcinoma (HCC) is one of the most common malignant tumors and is the third frequent cause of cancer-related death in the world [1]. Surgical treatments including liver transplantation (LT) and hepatic resection (HR), as well as medical treatments such as radiofrequency ablation (RFA) and transarterial chemoembolization (TACE), are widely performed for the treatment of HCC. In the Barcelona Clinic Liver Cancer staging and treatment strategy updated in 2011, HR is recommended for the treatment of single HCC < 3 cm, Child-Pugh A and B with performance status 0, and normal portal pressure/bilirubin [2]. LT is recommended for the treatment of very early (single HCC < 2 cm) and early stages (single HCC or 3 nodules < 3 cm with performance status 0) in patients with increased portal pressure/bilirubin and without associated diseases [2]. The Japanese treatment algorithm for HCC recommends HR for Child-Pugh A and B patients with 3 or few tumors irrespective of tumor size [3]. In contrast, LT is recommended for Child-Pugh C patients within the Milan criteria.

In the clinical setting, however, the indications for HR and LT are not definitely separated or defined. For example, not a few transplant centers in the world use expanded transplantation criteria for HCC beyond the Milan criteria, including the University of California San Francisco criteria, Kyoto criteria, and Tokyo criteria [4–9]. Regarding liver function, LT is sometimes performed for Child-Pugh A and B patients who cannot undergo HR or RFA due to liver dysfunction or tumor location. As for type of LT, especially in Japan, living donor LT (LDLT) is usually performed for such patients in whom HR or RFA is not indicated due to the shortage of deceased donors. Therefore, a comparison of outcomes after these surgical therapies is needed to validate the above algorithms and our clinical decision-making. However, there has been no study to compare outcomes between well-matched groups after LDLT and HR for HCC.

It is clinically impossible to perform a randomized, controlled trial to compare the usefulness of LDLT and HR for HCC. In the present study, therefore, outcomes after LDLT or HR for HCC were retrospectively examined in the country where LDLT is mainstream. The technique of propensity score computer-matching of preoperative risk factors was used to obtain a valid comparison between the 2 surgical treatment groups in all patients and in patients within our expanded LT criteria for HCC incorporating biomarker for HCC.

## 2. Patients and Methods

### 2.1. Study Patients

A total of 732 patients and 204 patients underwent HR and LDLT, respectively, for HCC at Kyoto University Hospital between January 1999 and August 2012 (Table 1). Patients with vascular invasion on preoperative imaging, including computed tomography (CT) and magnetic resonance imaging (MRI), distant metastasis, and Child-Pugh classification C and those who lacking data for tumor markers such as alpha-fetoprotein (AFP) and des-gamma-carboxy prothrombin (DCP) and exact preoperative tumor size or number were excluded. The remaining 735 patients (107 patients who underwent LT and 628 patients who underwent HR) were included in this study. We also defined the 415 patients with our new expanded criteria for LT for HCC (the Kyoto criteria). Patient records/information was anonymized and deidentified prior to analysis. The study was approved by the Ethics Committee of Kyoto University and conducted in accordance with the Declaration of Helsinki of 1996.

### 2.2. Surgical Procedures, Surgical Indications, and Postoperative Follow-Up for Hepatic Resection

Among the 732 patients who underwent HR, the surgical procedures consisted of 287 partial resections, 63 segmentectomies, 143 sectionectomies, 220 bisectionectomies, and 19 trisectionectomies. Segments were defined according to Couinaud's classification system [10]. The indications for liver resection were based on platelet count, total bilirubin level, prothrombin time, CT volumetry, and the indocyanine green Krem index (ICGkrem), which is calculated by multiplying the ICG disappearance rate by the ratio of remnant liver volume to the total liver volume, using an ICGkrem of 0.03 as the lower limit for surgery. In cases of ICGkrem < 0.03, the results of liver scintigraphy using technetium-99m-labeled asialoglycoprotein analog were also considered. Following discharge from hospital, the patients underwent clinical follow-up (including a blood test and CT scan) every 3 months. When abnormal data were obtained or suspected lesions were detected, further examinations including contrast ultrasonography, MRI, or positron emission tomography were performed.

### 2.3. Surgical Procedures, Indications, and Immunosuppressive Treatments for LT

As for LT, the selection criteria for the recipients, as well as the surgical techniques for the donor and recipient, have been described in detail elsewhere [11–13]. Until December 2006, our primary institutional selection criteria for LDLT for HCC included any size or number of tumors, provided that there was no distant metastasis or gross vascular involvement on preoperative imaging. Since January 2007, we have been using the Kyoto criteria as described elsewhere [9]. Briefly, risk factors for recurrence were analyzed in 136 patients who underwent LT for HCC until December 2006. Based on the results of this study, we established the Kyoto criteria combining three independent significant risk factors for recurrence, including tumor number and tumor size based on the findings of pretransplant imaging and tumor markers: tumor number ≤ 10, maximal diameter of each tumor ≤ 5 cm, and serum DCP levels ≤ 400 mAU/mL. DCP, also known as protein induced by vitamin K absence or antagonist II, is a well-known tumor marker of HCC whose expression is significantly correlated with poor prognosis [14–17].

Orthotopic LDLT was performed using a left lobe graft for 24 patients and a right lobe graft for 180 patients. Preoperative imaging showed that 124 patients met the Milan criteria and 80 did not meet the Milan criteria, and 154 patients met the Kyoto criteria and 50 did not meet the Kyoto criteria. Preoperative measurement of serum DCP and AFP levels has been described in detail elsewhere [18].

The standard immunosuppression protocol consisted of tacrolimus and low-dose steroid [19, 20]. No patients were switched to mammalian target of rapamycin inhibitor.

### 2.4. Statistical Analysis

We conducted the statistical analysis for all the defined patients (*n* = 735) and subgroup patients with Kyoto criteria (*n* = 415). The survival rates and recurrence rate of patients who underwent LT or HR were calculated from the date of operation.

Because this study was nonrandomized and observational, potential confounding selection bias was accounted for with propensity score analysis. We computed the propensityscore by using multiple logistic regression with the dependent variable receiving HR or LT. The independent variables were age, sex, Child-Pugh classification, tumor number, tumor size, AFP, and DCP. We were able to match 40 patients undergoing LT to 40 patients undergoing HR. For Kyoto criteria patients, we match 23 patients undergoing LT to 23 patients undergoing HR. For the propensity score-matched sample, patient characteristics of the HR and LT groups were compared using McNemer's test for binominal categorical variables, Mantel's *χ* ^2^ test for multinomial categorical variables, and paired *t*-test for continuous variables. For evaluating the association between outcomes (survival and recurrence) and therapy (LT or HR), odds ratio was estimated by using the conditional logistic regression. Cumulative overall survival and recurrence rates were calculated using Kaplan-Meier methods, and differences between curves were evaluated using the log-rank test.

A *P* value < 0.05 was considered significant. All statistical data were generated using JMP 5.0.1 and SAS 9.2 (SAS Institute, Cary, NC) and Prism 5 (GraphPad Software, Inc., La Jolla, CA).

## 3. Results

Table 2 summarizes the baseline characteristics of the two propensity score-matched groups. Both groups were well balanced for all variables, including age, sex, the maximum diameter of the tumor, number of tumors, DCP level, AFP level, and Child-Pugh classification. There were no significant differences in the baseline data between the groups. The median follow-up time in the HR group and the LT group was 87 months and 84 months, respectively.

**Figure 1:**
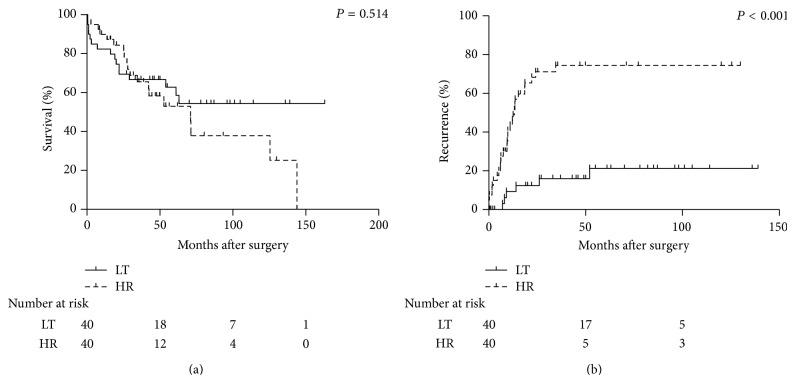
Overall survival rates (a) and recurrence rates (b) in the HR and LT groups after adjustment with propensity scores in all patients. HR, hepatic resection; LT, liver transplantation.

The 1- and 5-year overall survival rates were 90% and 53% in the HR group and 82% and 63% in the LT group, respectively. They were not significantly different between the two therapies (*P* = 0.514) (Figure 1(a)). The odds ratio using the propensity score-matched method was 0.842 (95% confidence interval 0.433–1.638; *P* = 0.613). In contrast, the 1- and 5-year recurrence rates were significantly lower in the LT group (9% and 21%) than in the HR group (43% and 74%) (*P* < 0.001) (Figure 1(b)). The odds ratio using the propensity score-matched method was 0.214 (95% confidence interval 0.089–0.518; *P* = 0.001).

**Figure 2:**
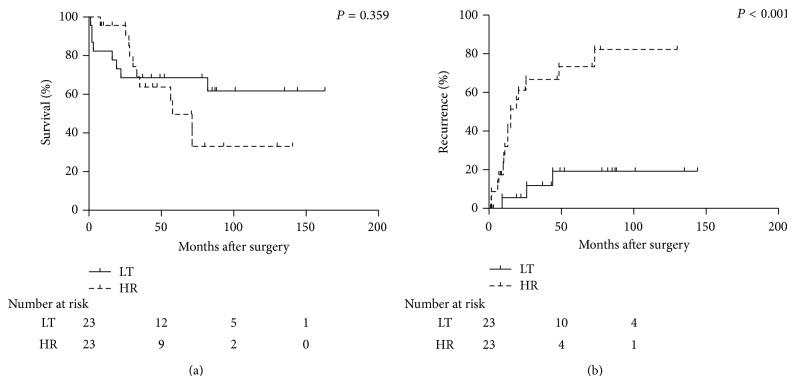
Overall survival rates (a) and recurrence rates (b) in the HR and LT groups after adjustment with propensity scores in patients within the Kyoto criteria. HR, hepatic resection; LT, liver transplantation.

Next, a similar analysis was performed in the 46 propensity-matched patients within our new expanded criteria for LT for HCC (the Kyoto criteria). The baseline characteristics of the two propensity-matched groups are shown in Table 3. Both groups were well balanced for all variables, including age, sex, the maximum diameter of the tumor, number of tumors, DCP level, AFP level, and Child-Pugh classification. The 1- and 5-year overall survival rates were 96% and 50% in the HR group and 82% and 68% in the LT group, respectively. There were no significant differences between the two therapies (*P* = 0.359) (Figure 2(a)). The odds ratio was 0.727 (95% confidence interval 0.293–1.808; *P* = 0.493). In contrast, the 1- and 5-year recurrence rates were significantly lower in the LT group (5% and 18%) than in the HR group (31% and 74%) (*P* < 0.001) (Figure 2(b)). The odds ratio was 0.200 (95% confidence interval 0.058–0.691; *P* = 0.010).

The causes of death are quite different between the two groups. In the HR group, 16 of 19 patients (84%) and 9 of 11 patients (82%) died of HCC recurrence in each propensity score analysis. In contrast, only 4 of 16 (25%) and 2 of 8 (25%) died of HCC recurrence in the LT group in each propensity score analysis. Most of the remaining patients died of noncancerous causes including sepsis and graft failure in the early post-LT period.

## 4. Discussion

To the best of our knowledge, this study is the first to compare outcomes between well-matched groups after HR and after LDLT for HCC using Cox proportional hazards model analysis at a single institution. It was clearly demonstrated that patients who underwent LDLT had significantly lower recurrence rates than those who underwent HR, while a survival benefit could not be shown in the LDLT group. So far, not a few studies have tried to compare outcomes after HR and LT [21–27]. However, most studies have used a heterogeneous group of patients in relation to tumor burden, as well as underlying liver dysfunction. In contrast, the present study compared survival and recurrence rates of HR versus LT for HCC between well-matched groups, including Child-Pugh classification, tumor number, tumor size, AFP, and DCP levels. Dhir et al. recently conducted a meta-analysis of 10 published reports to compare survival outcomes after LT and HR in patients with early HCC, namely, within the Milan criteria, and well-compensated cirrhosis [28]. The meta-analysis of all 10 published studies revealed a survival advantage for LT (odds ratio 0.581; *P* = 0.027). However, analysis of intention-to-treat studies only found no significant difference (odds ratio 0.600; *P* = 0.166). Most recently, Proneth et al. reported that no survival advantage of LT could be found compared with HR in a meta-analysis by use of seven studies with a total of 1572 patients [29]. Taken together with these findings, the survival advantage of LT is unclear, whereas LT evidently offers a significantly better recurrence rate than HR.

LT is well known to have noteworthy advantages that HR does not possess in the treatment of HCC. LT can treat not only intrahepatic tumors but also underlying liver diseases. On the other hand, LT has crucial disadvantages that HR does not have. The greatest disadvantage of LT is the relatively high mortality in the early post-LT period. Lee et al. compared early operative mortality in patients undergoing LT (*n* = 78) and HR (*n* = 130) with HCC and underlying Child-Pugh A or B cirrhosis [25]. They reported that the early operative mortality rate was higher in the LT group (5.1%) than in the HR group (0.8%), in line with our previous report that the mortality rate in all patients who underwent LT in 2007 was 4.8% [30]. In the present study, the sharp decline of the survival curves in the LT group was shown in both well-matched groups in all patients (Figure 1(a)) and in patients within the Kyoto criteria (Figure 2(a)). In contrast with the HR group, the major causes of death in the LT group were noncancerous reasons including sepsis and graft failure, especially in the early post-LT period. However, recent advances in perioperative management and technical innovations have gradually decreased the in-hospital mortality rate. If the mortality risk after LT could be diminished to that after HR, the overall survival rates in the LT group would become higher than those in the HR group due to the low risk of HCC recurrence. The next crucial disadvantage of LT is low organ availability with both living and deceased donor LT. Donor shortage is a common problem in Western and Eastern countries. Moreover, the dropout rate while waiting for deceased donor LT is not negligible. Literally, the total dropout rate at 12 months after listing for LT ranges from 11% to 30% [21, 31–33]. These downsides associated with LT reduce its viability as an alternative treatment option for patients with HCC.

In the present study, 46 propensity-matched patients within our new expanded criteria for LT for HCC, the Kyoto criteria, were further analyzed. The meta-analysis by Dhir et al. examined patients with early HCC within the Milan criteria only [28]. In contrast, this analysis included patients with advanced HCC in size and tumor number. Interestingly, LT showed significantly lower recurrence rates than HR even in patients with more advanced HCC compared with the Milan criteria, although no survival benefit was shown in this subgroup analysis. We have so far reported the usefulness of the Kyoto criteria as expanded selection criteria with a low recurrence rate [9, 34, 35]. The important point of the Kyoto criteria is incorporating DCP, a surrogate marker of tumor aggressiveness, as a variable to exclude patients with a high risk for recurrence preoperatively. Furthermore, the findings of the current study support the usefulness of DCP. Compared to our previous report that showed 5-year recurrence rates less than 5% in patients within the Kyoto criteria [35], however, the 5-year recurrence rate of 18% in the LT group in the present study is somewhat higher. Taking into consideration the issue of organ shortage, HR might be the first choice for these propensity-matched patients with HCC and underlying Child-Pugh A or B cirrhosis, in line with the concept of salvage transplantation [36].

Some limitations must be borne in mind when considering this study. First, the patient number included in this propensity-matched cohort was small, even though our institute is the largest LT center in Japan. In Japan, LT is usually selected for patients with Child-Pugh classification C, while HR is selected for patients with Child-Pugh classification A or B. Moreover, patients with vascular invasion on preoperative imaging were excluded from the study due to this being a contraindication for LT. These factors led to small patient numbers in the propensity-matched cohort. Therefore, a multicenter, nationwide study is needed to confirm the present findings. Second, the analysis used in this study was not an intention-to-treat analysis. Dhir et al. emphasized that patients who demonstrated disease progression while on the waiting list and became unsuitable for transplantation or died during waiting should be included to compare outcomes of LT and HR in patients with HCC [28]. In the present study, however, all patients in the LT group underwent LDLT, resulting in quite a low dropout rate. Furthermore, 28 of 40 patients in the LT group had a history of pretreatment including TACE, RFA, and HR. Moreover, patients in the HR group also received TACE or RFA after HCC recurrence. Strictly speaking, the survival rate after LT or HR for HCC should be compared between patients with no history of pretreatment and no postoperative treatment for recurrent lesions. In other words, usual comparison of outcomes after LT and HR does not exactly reflect the efficacies of LT and HR themselves. To accurately compare outcomes after LT or HR, a randomized, controlled study is needed; however, it is actually impossible and not ethically appropriate. Therefore, the propensity score-matching analysis used in the present study could be an alternative to a randomized, controlled study. Finally, the period of patient enrollment varied from January 1999 to August 2012. Surgical techniques, perioperative management, diagnostic imaging, and treatment modalities for HCC recurrence have improved dramatically in this decade for both HR and LT. Therefore, to exclude such bias, we should compare patients who underwent each surgical therapy over several years. In this context, a multicenter, nationwide study would be better to obtain definite conclusions.

## 5. Conclusions

LDLT can provide significantly better recurrence rates than HR based on this propensity score-matching study in all patients as well as those within our expanded selection criteria incorporating biomarker for HCC, although no survival benefit was seen in the LDLT group due to noncancerous causes of death in the early post-LT period. Therefore, HR should be considered a valid alternative to LDLT taking into consideration the risk for the living donor at present. Efforts to solve the problems associated with LT, including relatively high post-transplant mortality and morbidity rates and donor shortages, are needed to take maximum advantage of the merits of LDLT to improve the treatment of HCC.

## Figures and Tables

**Table 1:** Baseline characteristics of all patients who underwent HR and LDLT.

Characteristic	LT (*n* = 204)	HR (*n* = 732)	*P* value
Age (years)	57 (22–69)^∗^	67 (20–90)	<0.001
Sex (male/female)	140/64	576/156	0.004
Child-Pugh classification (A/B/C)	30/78/96	675/55/2	<0.001
DCP (mAU/mL)	50 (5–20600)^∗^	164 (6–355000)^∗^	0.002
AFP (ng/mL)	33 (0.9–212220)^∗^	21.9 (0.9–360093)^∗^	0.660
Maximum tumor size (cm)	4.5 (0.5–10.0)	4.0 (0.7–25.0)	<0.001
Number of tumors	2 (1–186)^∗^	1 (1–15)^∗^	<0.001

^∗^Data are given as median (range).

Tumor characteristics are those found on the preoperative imaging.

DCP, des-gamma-carboxy prothrombin; AFP, alpha-fetoprotein.

**Table 2:** Baseline characteristics of the two propensity-matched groups in all patients.

Characteristic	LT (*n* = 40)	HR (*n* = 40)	*P* value
Age (years)	60 (23–69)^∗^	58 (31–69)	0.671
Sex (male/female)	29/11	29/11	1.000
Underlying liver disease			0.784
Viral hepatitis C	24	22	
Viral hepatitis B	12	12	
Others	4	6	
Child-Pugh classification (A/B)	19/21	19/21	1.000
DCP (mAU/mL)	37 (5–6740)^∗^	55.5 (9–51286)^∗^	0.120
AFP (ng/mL)	34.1 (0.9–3284)^∗^	35.4 (1.7–107579)^∗^	0.301
Maximum tumor size (cm)	3.1 (1.0–12.0)	3.4 (0.7–14.5)	0.638
Number of tumors	2 (1–100)^∗^	1 (1–15)^∗^	0.250
Milan criteria met	22 (55%)	26 (65%)	0.361
Postoperative mortality	6 (15%)	2 (5%)	0.136

^∗^Data are given as median (range).

Tumor characteristics are those found on the preoperative imaging.

DCP, des-gamma-carboxy prothrombin; AFP, alpha-fetoprotein.

Postoperative mortality means postoperative death within 90 days after surgery in this analysis.

**Table 3:** Baseline characteristics of the two propensity-matched groups within the Kyoto criteria.

Characteristic	LT (*n* = 23)	HR (*n* = 23)	*P* value
Age (years)	58 (27–68)^∗^	57 (42–69)	0.811
Sex (male/female)	15/8	15/8	1.000
Underlying liver disease			0.194
Viral hepatitis C	14	16	
Viral hepatitis B	9	5	
Others	0	2	
Child-Pugh classification (A/B)	10/13	9/14	0.765
DCP	30 (5–266)^∗^	44 (9–277)^∗^	0.150
AFP	29 (3–28074)^∗^	28 (3–1321)^∗^	0.725
Maximum tumor size (cm)	1.8 (0.8–5.0)	2.1 (0.9–5.0)	0.349
Number of tumors	1 (1–5)^∗^	1 (1–5)^∗^	0.779
Milan criteria met	18 (78%)	21 (91%)	0.218

^∗^Data are given as median (range).

Tumor characteristics are those found on the preoperative imaging.

DCP, des-gamma-carboxy prothrombin; AFP, alpha-fetoprotein.
